# Cervical cancer screening among incarcerated women

**DOI:** 10.1371/journal.pone.0199220

**Published:** 2018-06-26

**Authors:** Patricia J. Kelly, Molly Allison, Megha Ramaswamy

**Affiliations:** 1 University of Missouri-Kansas City, Kansas City, Missouri, United States of America; 2 University of Kansas School of Medicine, Kansas City, Kansas, United States of America; University of North Carolina at Chapel Hill, UNITED STATES

## Abstract

**Background:**

Women with a history of incarceration bear a disproportionate burden of cervical disease and have special characteristics that affect their intent and/or ability to adhere to cervical screening and follow-up recommendations. The goal of this study was to identify factors associated with cervical cancer screening and screening outcomes among incarcerated women.

**Methods:**

We applied a framework of predisposing factors, enabling factors and population-specific characteristics that could impact screening behaviors and outcomes for this population. We used bivariate chi-square tests and Wilcoxon signed-rank tests to analyze data previously collected from 290 incarcerated women.

**Results:**

Cervical cancer screening belief score, as a predisposing factor, was associated with women who had an up-to-date Pap test and who had a cervical cancer diagnosis ever in their life. Both a sexual history containing high-risk behaviors and a history of abuse, population–specific factors, were each associated with having had an abnormal pap; mental health, incarceration, and substance use histories were each associated with having a diagnosis of cervical cancer.

**Conclusions:**

The significant differences in outcomes for these population-specific factors suggest the need for a health services approach that addresses the challenges to the cervical cancer preventive health needs of incarcerated women.

**Implications for practice:**

Providers working with vulnerable populations such as women who have been incarcerated should be aware that their risk histories have an influence on their follow-up behaviors. These women will need extra support for cervical cancer screening and follow-up care.

## Background

Cervical cancer is the result of infection with one of the known carcinogenic subtypes of the human papillomavirus (HPV),[1] and is one of the few cancers for which screening can have a major impact on prevention. Access to screening and appropriate follow-up of positive tests can eliminate disease in individuals and decrease mortality for populations.[1] In addition, vaccines for HPV can significantly reduce the rate of occurrence of this disease.[2] Incarcerated women in North America are four to five times more likely to report a cervical cancer diagnosis compared to the general population of women, and this is in large part a result to the challenges that these women face in receiving appropriate follow-up when they are screened.[3–4]

Certain demographic characteristics, including low income, low educational attainment, and minority status, are known barriers to cervical cancer screening.[5–6] Women’s self-perception of their susceptibility to cancer, and their cultural beliefs about cancer, have both been shown to be barriers to cervical cancer screening, and are both susceptible to change with research-based interventions.[7] Women with a criminal justice history, in itself a risk factor for cervical cancer, disproportionately share the interrelated risk factors of substance use, mental health problems, sexual risk behaviors, and histories of physical and sexual abuse.[8]

Several authors have suggested that research findings on screenings in general, and on cervical cancer screening in particular, tend over-estimate screening. Thus, they do not provide a true picture of the disparities in access that make it less likely for women in vulnerable populations to be screened.[9, 10] In addition to forgetfulness and the tendency to provide socially desirable responses, women with low cervical health literacy often equate all pelvic exams with Pap smears, resulting in over-reporting. [11]The misreporting of Pap smear histories obfuscates the full picture of women’s true cervical cancer risk.

Our goal was to identify factors associated with cervical cancer screening and screening outcomes among incarcerated women, keeping in mind the limitations cited above. We adapted Andersen’s updated behavioral model for health care use, which has been previously applied in criminal justice settings. [12] We hypothesized three broad categories that contribute to personal health practices about cancer screening and outcomes:

- Predisposing factors: demographics and health beliefs,
- Enabling factors: health insurance, housing and employment status, and receipt of public benefits,
- Population-specific factors: histories of substance use or childhood sexual abuse.^9^

The specific research questions in the current study using this model were: 1) Which of these factors are correlated with having had a Papanicolaou (Pap) test within the past three years? and 2) Which of these factors are correlated with having had an abnormal Pap test result or cervical cancer diagnosis?

## Methods

We conducted a secondary analysis of data previously collected over a six-month period in 2010 on the health needs and service usage of 290 women in three county jails in the Kansas City metropolitan area.^4^ During the study period, an estimated 50% of the women from the three facilities were interviewed. The sample was similar in age, race, and ethnic characteristics to the overall population of women in the three jails at the time. The study was approved by the Institutional Review Board at the University of Kansas Medical Center.

### Procedures

Women were continuously recruited into this study over a six-month period using flyers and word of mouth from jail staff. Interviewers read a standardized recruitment script and consent form to each potential participant and administered a face-to-face survey [S1 File] in a semiprivate space at the jail for those providing written consent. As compensation for participating, each woman was given a $5 credit to her commissary account or a gift basket worth $5.

### Measures

The dependent variable for the first research question, was operationalized as ever having an up-to-date Pap test, was assessed with the question, “How often do you usually have a Pap test?” Reponses of “Every 2–3 years” or “Every year” were considered as within the recommended three-year time frame for screening. The dependent variable for the second research question, operationalized as ever having had an abnormal Pap test result or cervical cancer diagnosis, was assessed with the questions: “Has a doctor, nurse, or other health professional ever told you that you had an abnormal Pap result?” and “Has a doctor, nurse, or other health professional ever told you that you had cervical cancer?”

For independent variables, we assessed predisposing factors, enabling factors and population-specific factors specific to women with a criminal justice history.

The predisposing factors we included in the analysis were both demographic and related to beliefs about cervical cancer screening. The demographic factors were age at interview, race, and education level. Beliefswere measured by eight statements modified from Champion’s health belief model for breast cancer:

1. Getting tested for cervical cancer will lead to better health,
2. I do not have time to get tested for cervical cancer,
3. Cervical cancer tests are disgusting,
4. Cervical cancer tests are uncomfortable,
5. Cervical cancer tests are embarrassing,
6. I am afraid a cervical cancer test would show I have cervical cancer,
7. I am concerned that cervical cancer testing would be painful, and
8. I am concerned about the cost of getting a cervical cancer test.

Responses were made on a four point Likert scale of “Strongly disagree,” “Disagree,” “Agree,” or “Strongly agree”^.^ [13] The instrument was pretested with a similar population and no adjustments were deemed necessary. One item (#1) was reverse scored; items were totaled and averaged. Higher scores indicated more barriers to cervical cancer screening and less-positive beliefs. The scale had a moderate reliability score (Cronbach’s alpha = 0.6).

Enabling factors were assessed with four closed-ended dichotomous questions on health insurance, housing and employment status, and receipt of public benefits.

Population-specific factors were assessed with five items:

1. Sexual history was assessed with two questions: “Have you ever been diagnosed with HIV/AIDS, syphilis, gonorrhea, or chlamydia?” and “How many sexual partners did you have in the three months prior to this incarceration?” Responses were dichotomized into participants with three or more partners and less than three partners. While many studies dichotomize responses to this question into one and more than one, we made this choice because 11% of our sample in previous work had sex with more than three partners.
2. Mental health history was assessed with one item, “Were you ever told by medical professional that you had depression, anxiety, schizophrenia, or bipolar disease?”
3. Incarceration history was assessed with two items: “How many months have you spent in jail/prison in your life?” and “How many days were you incarcerated in the past year?” This variable was dichotomized to greater than/equal to and less than six months, because six months was approximately the median value for participants’ lifetime months in jail/prison. Similarly, the number of days incarcerated in the past year was dichotomized to greater than/equal to or less than 36 days, because this was the median.
4. Three closed-ended dichotomous questions assessed abuse history:
  - Partner abuse was assessed with an adaption of the Verbal HITS Scale, “In the one year prior to incarceration, did a partner physically hurt, insult or scream at you on a regular basis or fairly often?” [14]
  - Childhood physical abuse was assessed with the Childhood Experiences of Violence Questionnaire, and considered positive if the participant responded that an adult hit, pushed/shoved, or kicked/punched her two or more times before age 16; [15]
  - Sexual abuse before age 16 was assessed with one item asking: “Did anyone ever do any of the following things when you didn’t want them to: touch the private parts of your body, make you touch their private parts, threaten or try to have sex with you, or sexually force themselves on you?”, and one or more times was considered a positive response. [15]
5. Substance use history was assessed two way. The first was the use of two dichotomous questions: “Were you ever diagnosed with alcoholism or a substance abuse problem?” and “Did you use methamphetamines, PCP, heroin, crack, or powdered cocaine in the 30 days prior to this incarceration?”. The second was the use of six items developed from DSM-IV criteria, such as, ‘‘Did you need to use more drugs to get the same high as when you first started using?” (Cronbach’s alpha = 0.86). Participants who answered ‘‘yes” to three out of six items were classified as drug- dependent.

### Analysis

SAS Studio version 3.4 was used for statistical analyses. Descriptive statistics were generated for all variables and univariate tests determined percentages of missing values for all variables; those with high percentages of missing data were eliminated. Wilcoxon signed-rank analyses were performed to evaluate differences in cervical cancer screening belief scores across outcome variables. Bivariate tests were performed to determine associations with the outcome variables: up-to-date Pap test (“Every year” or “Every 2–3 years”), abnormal Pap test result, and cervical cancer diagnosis. For the cervical cancer diagnosis outcome, only women who had reported ever having an abnormal Pap test were included in analysis. Chi-square test statistics, corresponding p-values, and 95% confidence limits were used to verify significant associations. Bivariate tests with cell counts of less than 5 were analyzed with Likelihood Ratio Chi-square values. Multivariable regression models for each outcome were obtained using backward elimination of non-significant independent variables. Independent variables were kept in the models based on individual significance, overall significance of the model, and absence of multicollinearity among variables.

## Results

The average age of the 290 women in the sample population was 33.9 years (range 17–62, SD = 9.7). The majority self-identified as either White (43.0%) or Black (40.5%) and 70.1% had at least a high school education. Over half had received a mental health diagnosis (62%), met drug dependence criteria (55.1%), or experienced physical and/or sexual abuse before age 16 (64.4%). The majority of participants (77.2%) had had a Pap test in the last three years, and 40% of participants had received an abnormal Pap test result in their lifetime. Table 1 describes the frequencies of these and other *predisposing*, *enabling* and *population-specific factors*, as well as the dependent variables.

**Table 1:** Characteristics of sample, N = 290.

Theoretical Factors	Variables	N/%
Predisposing factors	Age, mean (range, SD)	33.9 (17–62; 9.7)
	Race/Ethnicity	
	White, Non-Hispanic	123/43.0%
	African-American, Non-Hispanic	116/40.5%
	Hispanic	24/ 8.3%
	Other	25/ 8.0%
	Education	
	Less than high school	86/29.8%
	High school or higher	202/70.1%
	Cervical cancer screening beliefs: Mean (range, SD)	2.15 (1.0–3.37; 0.34)
Enabling factors	Pre-incarceration insurance coverage	126/45.1%
	Public benefits before incarceration	133/54.9%
	Working pre-incarceration	107/37.6%
	Homeless at time of arrest	30/10.5%
Population-specific-factors	Sexual history	
	Lifetime history of STIs	58/20.3%
	More than 3 sex partners in past 3 months	32/11.2%
	Age at first sex, ≤ 15	156/53.7%
	Mental health history	
	Ever diagnosed with depression, anxiety, schizophrenia, or bipolar disease	178/62.0%
	Incarceration history	
	Lifetime months incarcerated mean (*SD*)	17.8 (31.3)
	Days incarcerated in the past year, mean (SD)	82.9 (96.5)
	Abuse history	
	Physically abused before age 16	172/59.3%
	Sexually abused before age 16	95/34.9%
	Abused in any way before age 16	187/64.4%
	Physically abused by partner in year before incarceration	127/46.3%
	Abused a partner in year before incarceration	81/29.6%
	Substance use history	
	Ever diagnosed with drug/alcohol problem	123/43.4%
	Hard drug use 30 days before incarceration	132/47.3%
	Drug dependent	128/55.1%
Personal health practices	Up-to-date Pap test	224/77.2%
	Up-to-date Pap test/no abnormal Pap test result/no cervical cancer diagnosis	116/40.0%
	Lifetime history of abnormal Pap test result	115/40.9%
	Lifetime history of cervical cancer diagnosis	29/10.9%

The average score on the cervical cancer screening beliefs scale (an enabling factor) was 2.15 (range 1.0–3.37; SD 0.34). Women who reported having an up-to-date Pap test (224/77.2%) had significantly lower (more positive) mean scores for cervical cancer screening beliefs (p = 0.006). Women who had ever been diagnosed with cervical cancer had significantly higher (more negative) mean scores for cervical cancer screening beliefs than women who had never been diagnosed (p = 0.02) (Table 2).

**Table 2:** Relationships between cervical cancer screening beliefs and dependent variables using Wilcoxon signed-rank tests^a^.

	N/%	Mean/SD	Normal z	Two-sided P> Z
Up to date Pap (N = 203)			2.70	0.006*
Yes	159/78.3%	96.1 (342)		
No	44/21.6%	123.1 (342)		
Abnormal Pap test history (N = 197)			1.35	0.17
Yes	79/40.1%	105.6 (390)		
No	118/59.8%	94.5 (390)		
History of cervical cancer diagnosis (N = 79)			1.73	0.08
Abnormal Pap with cervical cancer dx	22/27.8%	47.1 (91)		
Abnormal Pap, no cervical cancer dx	57/72.1%	37.2 (91)		

*p ≤ 0.05

**p ≤ 0.01

***p ≤ 0.001

^a^Only women who completed cervical cancer screening beliefs questions were included in this analysis.

N values for each dependent variable are reflected accordingly.

Table 3 summarizes significant associations found between the independent variables and the dependent variable of having an up-to-date Pap test, a history of abnormal Pap results, and a history of cervical cancer. Having an up-to-date Pap test was associated with sexual abuse before the age of 16 (OR 2.09, CI 1.08–4.03, p≤ .05). Having a history of an abnormal Pap test (OR 2.22, CI 1.36–3.61, p≤ .001), was significantly associated with race, with more than half of White women (51%) having an abnormal Pap history, compared to 34% of Black women and 27% of women in other racial groups. A history of an abnormal Pap test was also associated with four population-specific characteristics: a lifetime history of STIs (OR 1.90, CI 1.05–3.44, p≤ 0.05), physical abuse before the age of 16 (OR 1.82, CI 1.11–2.99, p≤ 0.05), partner abuse in the past year (OR 2.45, CI 1.48–4.04, p ≤ .001), and initial sex at or before age 15 (OR 1.77, CI 1.09–2.88, p≤0.05). A history of cervical cancer was significantly associated with race, with 5% of Black women ever having cervical cancer, compared to 17% of White women and 10% of women in other racial groups. A cervical cancer diagnosis was also associated with three population-specific characteristics: a positive mental health history (OR 3.87, CI 1.23–12.19, p ≤ 0.01), incarceration for six months or longer (OR 0.39, CI 0.16–0.97, p≤0.05), and hard drug use in the 30 days prior to the current incarceration (OR 2.46, CI 1.00–6.11, p≤0.05).

**Table 3:** Unadjusted odds ratios of variables with significant associations to cervical cancer screening outcomes, *N* = 290.

Individual independent variables	Up-to-date Pap test OR (95% CI)	History of abnormal Pap test OR (95% CI)	History of cervical cancer diagnosis^a^ OR (95% CI)
Demographics			
White	0.58 (0.33, 1.02)	2.22 (1.36, 3.61)**	2.06 (0.83, 5.07)
Black	1.48 (0.83, 2.62)	0.63 (0.38, 1.03)	0.36 (0.13,0.98)*
Population Characteristics			
Sexual			
Lifetime history of STIs	2.09 (0.93, 4.68)	1.90 (1.05, 3.44)*	1.07 (0.42, 2.78)
Age at first sex ≤15	1.12 (0.64, 1.95)	1.77 (1.09, 2.88)*	1.99 (0.79, 5.03)
Mental health history	0.91 (0.51, 1.61)	1.63 (0.98, 2.71)	3.87 (1.23, 12.19)*
Incarceration history			
Lifetime incarceration ≥6 mos.	0.65 (0.37, 1.13)	0.91 (0.56, 1.47)	0.39 (0.16, 0.97)*
Abuse history			
Sexual abuse before age 16	2.09 (1.08, 4.03)*	1.42 (0.85, 2.37)	0.96 (0.38, 2.37)
Physical abuse before age 16	1.28 (0.74, 2.24)	1.82 (1.11, 2.99)*	1.05 (0.42, 2.62)
Partner abuse in past year	0.82 (0.47, 1.44)	2.45 (1.48, 4.04)***	1.56 (0.62, 3.87)
Substance use history			
Hard drug use 30 days pre-incarceration	0.86 (0.49, 1.52)	1.43 (0.88, 2.33)	2.46 (1.00, 6.11)*
Insurance status			
-Insured prior to incarceration	1.55 (0.87, 2.75)	0.82 (0.50, 1.34)	0.97 (0.40, 2.32)

*p ≤ 0.05

**p ≤ 0.01

***p ≤ 0.001

^a^Cervical cancer diagnosis dichotomized by 1 = received an abnormal Pap test result with a cervical cancer diagnosis and 0 = received an abnormal Pap test result with no cervical cancer diagnosis.

Tables 4–6 display multivariable models for each health outcome. A combination of predisposing factors, enabling factors and population-specific factors contributed to these outcomes. The model for having an up-to-date Pap test included White race (OR 0.50, CI 0.27–0.90) had a negative association with having an up-to-date Pap test, while a history of sexual abuse (OR 2.23, CI 1.13–4.38) was positively associated with having an up-to date Pap test (when controlling for prior insurance status) (Table 4). The model for having an abnormal Pap test result included White race (OR 2.24, CI 1.34–3.73) and partner abuse in the year before incarceration (OR 2.40, CI 1.44–4.00) (Table 5). Finally, the model for having had a cervical cancer diagnosis was negatively associated having a mental health diagnosis (OR 1.24, CI 1.24–12.76) and a lifetime incarceration of 6 months or more (0.38, CI 0.15–0.97) (Table 6).

**Table 4:** Logistic regression model for dependent variable up-to-date Pap test, N = 290^a^.

Independent variable	ß	SE	χ^2^	P-value	OR (95% CI)
Predisposing factors					
White race	-0.68	0.30	5.16	0.02	0.50 (0.27, 0.90)
Population-specific factors					
Sexual abuse	0.80	0.34	5.47	0.01	2.23 (1.13, 4.38)
Enabling factors					
Insured before incarceration	0.57	0.31	3.35	0.06	1.77 (0.96, 3.26)

^a^Number of observations read = 290, number of observations used = 262.

Twenty-eight observations deleted due to missing values for response or explanatory variables.

**Table 5:** Logistic regression model for dependent variable abnormal Pap test result, N = 290^a^.

Independent variable	ß	SE	χ2	P-value	OR (95% CI)
Predisposing factors					
White race	0.80	0.26	9.57	0.002	2.24 (1.34, 3.73)
Population-specific factors					
Partner abuse in year before incarceration	0.87	0.26	11.34	0.0008	2.40 (1.44, 4.00)

^a^Number of observations read = 290, number of observations used = 266.

Twenty-four observations deleted due to missing values for response or explanatory variables.

**Table 6:** Logistic regression model for dependent variable cervical cancer diagnosis, N = 290^a^.

Independent variable	ß	SE	χ2	P-value	OR (95% CI)
Population-specific factors					
Mental health diagnosis	1.38	0.59	5.43	0.01	3.98 (1.24, 12.76)
Lifetime incarceration > 6 mos.	-0.95	0.47	4.08	0.04	0.38 (0.15, 0.97)

^a^Number of observations read = 290, number of observations used = 110.

180 observations deleted due to missing values for response or explanatory variables.

## Discussion

The findings from this study demonstrate that two of the construct variables of the hypothesized model, predisposing factors and population-specific factors, were important for understanding cervical cancer behaviors and outcomes in this sample of incarcerated women.

The predisposing demographic factor of race found in this sample is at odds with national statistics that have consistently shown a higher incidence of cervical cancer among Black compared to White women, suggesting that being incarcerated changes the previously-observed nature of the racial disparity. More positive scores on the predisposing factor of screening beliefs were associated with having an up-to-date Pap and a history of cervical cancer, findings which are similar to those of researchers working in a variety of populations. [16]

The association of sexual behavior, a population-specific factor, with abnormal Pap tests and cervical cancer has both behavioral and physiologic components. Early onset of sexual activity is associated with a greater number of partners; when these behaviors occur while the cervical squamocolumnar junction is still immature, it can facilitate infection with HPV, the causative agent of cervical cancer.[17] Both sexual and physical childhood abuse have a strong influence on adult sexual behaviors, including early onset of sexual activity and having multiple sexual partners.[18]

Mental illness and substance abuse both limit the time and energy women are able to use for non-emergent health screening or follow-up care. [19–20] Some researchers have found that women with a history of sexual abuse are less likely to receive regular cervical cancer screening than women without such a history. However, our study found that women with this history were more likely to have an up-to-date Pap than national figures, with 78% of women in this study having had screening in the last five years.[16] This is consistent with the association in the literature between partner abuse and both screening behaviors and cervical cancer history being positive; that is, women experiencing partner violence have more health screenings than those without abuse histories. [21–22]

The population-specific factors that were significant independent variables in this study—sexual history, mental health diagnoses, greater number of days spent in incarceration, abuse history and substance use—were so strongly associated with women obtaining cervical health care and making their health a priority that they obviously play an important role, although they cannot be deemed causal. These factors can be considered a syndemic, that is, a confluence of multiple health and social issues that interact to result in disproportionate rates of disease in vulnerable populations.[23] The existence of these variables in the population of incarcerated women in general, and of this particular syndemic has been previously documented, but has not been specifically linked to disease behaviors or outcomes. [8]

There were limitations to this study. Self-reported answers to survey questions, especially Pap test frequency, cervical screening outcomes, other health outcomes, and health service utilization may be open to recall bias and not be as accurate as medical records or reports. However, our recent pilot study assessing the relationship between women’s self-reports, medical records and a cervical health literacy score found a significant association between high literacy scores and accurate self-report. [24] This pilot was an attempt to address previous criticisms that Pap tests were potentially over-reported; the addition of a cervical health literacy assessment to future research using women’s self-report of Pap testing may improve the validity of findings.

An additional limitation was that, while the effect of drug dependence on cervical cancer screening and outcomes was assessed, there was a fair percentage (26%) of data missing for this variable. Hard drug use in the 30 days prior to incarceration provided another avenue of assessing drug use, but this variable may not be indicative of long-term drug use. The absence of robust, standardized measures of mental health status and intimate partner violence raised other concerns.

Findings from this study add to the literature by providing preliminary evidence that risk factors for cervical cancer screening and screening outcomes among a sample of incarcerated women are distinctive, specifically pertaining to abuse history, drug use, and mental health problems. These findings also provide a basis for a more rigorous investigation of how these adverse conditions may affect women’s ability to navigate obtaining cervical health care and subsequent follow-up care. Public health interventions that can acknowledge and integrate these issues into screening and follow-up care could deliver promising outcomes for this population of women.

## Supporting information

S1 File Correlates cervical cancer survey instrument. (DOCX)
