# Supplementary material for: Cervical cancer screening among incarcerated women
Source: PLoS One. 2018 Jun 26;13(6):e0199220. doi: 10.1371/journal.pone.0199220 (PMC6019745; doi:10.1371/journal.pone.0199220)
Supplement: S1 File — (DOCX) [file pone.0199220.s001.docx]

**Facility** Johnson County **Today’s date** _____________

**Interviewer’s Initials** _____________

**HEALTH RESOURCE SURVEY OF MEN AND WOMEN IN THE JUSTICE SYSTEM**

**DEMOGRAPHIC INFORMATION FINAL AS OF 4/5/10 (JoCo 10/25/10)**

**1. How would you describe your gender?** 1. _____Female

2. _____Male

3. _____Other

7. _____Don’t know

8. _____Prefer not to Answer

9. _____Not Applicable

| **2. Which of the following best describes you?**  1. _____Caucasian (White)  2. _____African-American (Black)  3. _____Asian and Pacific Islander  4. _____American Indian or Alaska Native  5. _____Bi-Racial  6. _____Other _____________________  7. _____Don’t Know  8. _____Prefer not to Answer  9. _____Not Applicable | **3. Are you Hispanic?**  1. _____Yes  2. _____No  7. _____Don’t know  8. _____Prefer not to Answer  9. _____Not Applicable |
| --- | --- |

**4. What was your zip code prior to incarceration?** _____________ (##### OR 7/8/9)

**Please list your initials and the street number of your last address before you were incarcerated:** ____ / _____/ _____ / _____ / _____ / _____ (First initial, middle initial, last initial, street # digit 1, street # digit 2, street # digit 3, street # digit 4; if missing, e.g. no middle name, enter 0; if more street # digits at end, leave off)

**5. What is your birth year? (YY)** ___ ___ (OR 7/8)

| **6. Where were you living at the time of your arrest?**   1. _____Alone in house, apartment 2. _____With spouse and/or children 3. _____With other relatives 4. _____With friends or roommate(s) 5. _____With a significant other 6. _____In a shelter 7. _____From place-to-place 8. _____Homeless, the streets 9. _____In an institution 10. _____Other_____________________   7. _____Don’t Know  8. _____Prefer not to Answer  9. _____Not Applicable | **7. Where will you live when you leave jail?**   1. _____Alone in house, apartment 2. _____With spouse and/or children 3. _____With other relatives 4. _____With friends or roommate(s) 5. _____With a significant other 6. _____In a shelter 7. _____From place-to-place 8. _____Homeless, the streets 9. _____In an institution 10. _____Other_____________________   7. _____Don’t know  8. _____Prefer not to Answer  9. _____Not Applicable |
| --- | --- |

**8. How many children under age 18 lived with you before you were incarcerated?** __________________ (# OR 7/8/9)

**9. How many children under age 5 lived with you before you were incarcerated?** __________________ (# OR 7/8/9)

**9a. Have any of your children ever been in foster care system?**

1. _____ Yes
2. _____ No

7. _____Don’t know

8. _____Prefer not to Answer

**9b. Were you ever in the foster care system?**

1. _____ Yes
2. _____ No

7. _____Don’t know

8. _____Prefer not to Answer

**10. What is your marital or partner status?**

1. _____ Single
2. _____ Married
3. _____ Living with common law partner
4. _____ Living with same sex partner
5. _____ Separated
6. _____ Divorced
7. _____ Widowed

77. _____Don’t know

88. _____Prefer not to Answer

1. **What is the highest level in school that you have ever completed?**
2. _____Less than high school
3. _____Graduated high school/GED
4. _____Some college
5. _____College degree
6. _____Graduate degree or higher

7. _____Don’t know

8. _____Prefer not to Answer

9. _____Not Applicable

1. **What was your employment situation before you were incarcerated?**
2. _____Working at a regular job 35 hours a week or more
3. _____Working at a regular job part-time
4. _____Working on and off (not at a regular job)
5. _____Unemployed and looking for work
6. _____Unemployed and not looking for work
7. _____In school only
8. ____ Disabled, not able to work

77. _____Don’t know

88. _____Prefer not to Answer

99. _____Not Applicable

**12a. Have you ever served in the military, for example the National Guard, Reserves, Army, Navy, or Air Force?**

1. _____ Yes
2. _____ No

7. _____Don’t know

8. _____Prefer not to Answer

**HEALTH SERVICE UTILIZATION, MEDICAL HISTORY, AND REPRODUCTIVE HEALTH PRACTICES**

**In the past year, how many times did you…**

|  | Never  0 | Once  1 | More than once  2 | Don’t know  7 | Prefer not to Answer  8 | NA  9 |
| --- | --- | --- | --- | --- | --- | --- |
| 1. **Use an emergency room for medical problems or injuries?** |  |  |  |  |  |  |
| 1. **Get admitted to a hospital overnight or longer?** |  |  |  |  |  |  |
| 1. **Go to any sort of clinic for a checkup, medical problem, or an injury (not counting ER visits)?** |  |  |  |  |  |  |
| 1. **See a dentist?** |  |  |  |  |  |  |
| 1. **Receive substance abuse or alcohol abuse treatment?** |  |  |  |  |  |  |
| 1. **Receive treatment for a mental health problem?** |  |  |  |  |  |  |
| 1. **Go to any type of medical facility for a sexually transmitted infection-type problem?** |  |  |  |  |  |  |
| 1. **Go to any type of medical facility to get help preventing pregnancy or getting birth control?** |  |  |  |  |  |  |
| 1. **Go to any type of medical facility to get help with any other type of reproductive health condition, such as pregnancy?** |  |  |  |  |  |  |

**Show Response Card #1**

1. **If you said you used an emergency room, which hospital did you go to? (Enter number of facility below for up to 3 visits in past year)**

_____________ _____________ _____________

1. **If you said you were admitted to a hospital overnight or longer, which hospital did you go to? (Enter number of facility below for up to 3 visits in past year)**

_____________ _____________ _____________

1. **If you said you went to any sort of clinic for a checkup, medical problem, or an injury (not counting ER visits) which clinic did you go to? (Enter number of facility below for up to 3 visits in past year)**

_____________ _____________ _____________

1. **If you said you went to see a dentist, where did you go? (Enter number of facility below for up to 3 visits in past year)**

_____________ _____________ _____________

1. **If you said you received substance or alcohol abuse treatment, where did you go? (Enter number of facility below for up to 3 visits in past year)**

_____________ _____________ _____________

1. **If you said you treatment for any mental health problem, where did you go? (Enter number of facility below for up to 3 visits in past year)**

_____________ _____________ _____________

1. **If you said you went to a medical facility for a sexually transmitted infection-type problem, where did you go? (Enter number of facility below for up to 3 visits in past year)**

_____________ _____________ _____________

1. **If you said you went to a medical facility to get help preventing pregnancy or to get birth control, where did you go? (Enter number of facility below for up to 3 visits in past year)**

_____________ _____________ _____________

1. **If you said you went to a medical facility to get help with any other reproductive health condition, like pregnancy, where did you go? (Enter number of facility below for up to 3 visits in past year)**

_____________ _____________ _____________

1. **A personal doctor or nurse is a health provider who knows you the best. This can be a general doctor, a specialist doctor, a physician’s assistant, a nurse, or nurse practitioner. Do you have one person you think of as your personal doctor or nurse?**
2. ____ Yes
3. _____No

7. _____Don’t know

8. _____Prefer not to Answer

9. _____Not Applicable

**Have you ever been told by a doctor, dentist, physician's assistant, nurse, or nurse practitioner that you had…**

|  | Yes  1 | No  2 | Don’t know  7 | Prefer N Ans  8 | NA  9 |
| --- | --- | --- | --- | --- | --- |
| 1. **Hepatitis A** |  |  |  |  |  |
| 1. **Hepatitis B** |  |  |  |  |  |
| 1. **Hepatitis C** |  |  |  |  |  |
| 1. **HIV/AIDS** |  |  |  |  |  |
| 1. **Syphilis** |  |  |  |  |  |
| 1. **Gonorrhea** |  |  |  |  |  |
| 1. **Chlamydia** |  |  |  |  |  |
| 1. **High blood pressure** |  |  |  |  |  |
| 1. **Asthma** |  |  |  |  |  |
| 1. **Diabetes** |  |  |  |  |  |
| 1. **Cancer** |  |  |  |  |  |
| 1. **Dental problems** |  |  |  |  |  |
| 1. **Depression** |  |  |  |  |  |
| 1. **Anxiety** |  |  |  |  |  |
| 1. **Schizophrenia** |  |  |  |  |  |
| 1. **Bipolar disease** |  |  |  |  |  |
| 1. **Alcoholism** |  |  |  |  |  |
| 1. **Substance abuse problem** |  |  |  |  |  |
| 1. **Other problem**______________ |  |  |  |  |  |

**50a. Are you limited in any way in any activities because of physical, mental, emotional problems not having to do with being in jail?**

1. _____ Yes
2. _____ No

7. _____Don’t know

8. _____Prefer not to Answer

**50b. Do you now have any health problem that requires you to use special equipment such as a cane, a wheelchair, a special bed, or a special telephone?**

1. _____ Yes
2. _____ No

7. _____Don’t know

8. _____Prefer not to Answer

1. **Are you supposed to be taking any medicines now that someone prescribed to you?**
2. _____ Yes
3. _____ No

7. _____Don’t know

8. _____Prefer not to Answer

9. _____Not Applicable

1. **How do you pay for these medications?**
2. ____ Medicaid pays for it
3. _____I have other health insurance that pays for it
4. _____Cash, check, or credit card
5. _____I am not taking any because I could not pay
6. _____ I don’t use any medications
7. _____Other________________________

7. _____Don’t know

8. _____Prefer not to Answer

9. _____Not Applicable

1. **In the year before this incarceration, did you have any type of injury?**
2. _____Yes, from sports
3. _____Yes, from violence of some kind
4. _____Yes, from an accident
5. _____Yes, from something else
6. _____No injury

7. _____Don’t know

8. _____Prefer not to Answer

9. _____Not Applicable

1. **Have you or any of your former sex partners ever had a pregnancy that was not planned or was a surprise? Or was unexpected?**
2. _____Yes
3. _____No

7. _____Don’t know

8. _____Prefer not to Answer

9. _____Not Applicable

**55. Before you were incarcerated, how did you most often prevent pregnancy?**

**[Show Response Card #2**]

1. _____Condoms
2. _____Birth control pills
3. _____Other hormonal birth control methods (shot, the patch, the ring, IUD)
4. _____Withdrawal or “pulling out”
5. _____Tubal ligation or vasectomy
6. _____Not having sex during certain times of the month
7. _____Hope/prayer/luck
8. _____I didn’t use any methods to prevent pregnancy

77. _____Don’t know

88. _____Prefer not to Answer

99. _____Not Applicable

1. **Prior to your incarceration where did you or would you most likely go to get help with pregnancy prevention services? Pick the one place you either went to most often or would most likely go to.**
2. _____My family doctor
3. _____Health center or community clinic (not a city/county health department)
4. _____City or county health department
5. _____A hospital emergency room or clinic
6. _____Van (mobile health center)
7. _____Healer
8. _____Pharmacist
9. _____Store to buy condoms
10. _____Other health care provider_____________________

77. _____Don’t know

88. _____Prefer not to Answer

99. _____Not Applicable

1. **If you thought you had a sexually transmitted disease like gonorrhea (clap) or syphilis, where would you most likely go for treatment in the community? Pick the one place you would most likely go to.**
2. _____My family doctor
3. _____Health center or community clinic (not a city/county health department)
4. _____City or county health department
5. _____A hospital emergency room or clinic
6. _____Van (mobile health center)
7. _____Healer
8. _____Pharmacist
9. _____Other health care provider_____________________

77. _____Don’t know

88. _____Prefer not to Answer

99. _____Not Applicable

1. **Which of the following best described your health insurance coverage prior to incarceration?**
2. _____No health insurance
3. _____Private insurance
4. _____Medicaid
5. _____Medicare
6. _____VA
7. _____Some other kind of insurance
8. _____Other_____________________

77. _____Don’t know

88. _____Prefer not to Answer

99. _____Not Applicable

1. **Prior to your incarceration did you receive any of the following public benefits (check all that apply)?**
2. _____Food stamps
3. _____Disability
4. _____Social Security
5. _____Cash assistance/ TANF / AFDC

7. _____Don’t know

8. _____Prefer not to Answer

9. _____Not Applicable

**61. Have you ever exchanged sex for money, drugs, or life necessities?**

1. _____Yes
2. _____No

7. _____Don’t know

8. _____Prefer not to Answer

9. _____Not Applicable

**THESE NEXT THREE QUESTIONS ARE TO BE ASKED OF WOMEN, ONLY.**

**62. What age did you have sex for the first time?** __________________ (## OR 777/888/999)

**63. How many sex partners have you had in the three months prior to this incarceration?**

1. ____ None

2. _____One

3. _____Two-three

4. _____More than three

7. _____Don’t know

8. _____Prefer not to Answer

9. _____Not Applicable

**64. How many pregnancies have you ever had that you carried to full term?** __________________ (## OR 777/888/999)

**DRUG AND ALCOHOL USE**

**In the 30 days prior to this incarceration, have you used any of the following substances?**

|  | Yes  1 | No  2 | DK  7 | PrfrNAns  8 | NA  9 |
| --- | --- | --- | --- | --- | --- |
| 1. **Methamphetamines** |  |  |  |  |  |
| 1. **PCP** |  |  |  |  |  |
| 1. **Heroin** |  |  |  |  |  |
| 1. **Crack** |  |  |  |  |  |
| 1. **Powdered cocaine** |  |  |  |  |  |
| 1. **Marijuana** |  |  |  |  |  |
| 1. **Cigarettes (tobacco)** |  |  |  |  |  |

**Thinking about drug use in the year prior to incarceration…**

|  | Yes  1 | No  2 | Don’t Know  7 | Prefer N Ans  8 | NA  9 |
| --- | --- | --- | --- | --- | --- |
| **72. In the year prior to this incarceration did you need to use more drugs to get the same "high" as when you first started using?** |  |  |  |  |  |
| **73. Did you need to use more drugs than you wanted to?** |  |  |  |  |  |
| **74. Did you try to cut down drug use, but weren't able to?** |  |  |  |  |  |
| **75. Did drugs play a bigger role in your life than you wanted them to?** |  |  |  |  |  |
| **76. Did drug use cause you to give up or spend less time in school, work, with family or friends, or in recreational activities?** |  |  |  |  |  |
| **77. Did you ever keep using drugs even though it made you feel bad physically or emotionally?** |  |  |  |  |  |
| **78. Have you ever felt the need to cut down your drinking?** |  |  |  |  |  |
| **79. Have you ever felt annoyed by criticism of your drinking?** |  |  |  |  |  |
| **80. Have you ever had guilty feelings about drinking?** |  |  |  |  |  |
| **81. Have you ever taken a morning eye-opener?** |  |  |  |  |  |
| **82. Have you ever had a drinking problem?** |  |  |  |  |  |

**HISTORY OF VIOLENCE**

|  | Never  0 | 1-2 times  1 | 3-5 times  2 | 6-10 times  3 | > 10 times  4 | Don’t Know  7 | Prefer not to Answer  8 | NA  9 |
| --- | --- | --- | --- | --- | --- | --- | --- | --- |
| **In the year prior to your incarceration, did any of these things ever happen?** | | | | | | | | |
| **82a. Did a partner physically hurt you or insult or scream at you on a regular basis (or insult or scream at you fairly often)?** |  |  |  |  |  |  |  |  |
| **82b. Did you physically hurt a partner or insult or scream at this person on a regular basis (or insult or scream at them fairly often)?** |  |  |  |  |  |  |  |  |
| **Before age 16, how many times did any of the following things ever happen…** | | | | | | | | |
| 1. **Did an adult slap you on the face, head or ears or hit or spank you with something like a belt, wooden spoon or something hard?** |  |  |  |  |  |  |  |  |
| 1. **Did an adult push, grab, shove or throw something at you to hurt you?** |  |  |  |  |  |  |  |  |
| 1. **Did an adult kick, bite, punch, choke, burn you, or physically attack you in some way?** |  |  |  |  |  |  |  |  |
| 1. **Did anyone ever do any of the following things when you didn’t want them to: touch the private parts of your body, make you touch their private parts, threaten or try to have sex with you, sexually force themselves on you?** |  |  |  |  |  |  |  |  |

**CURRENT VIOLENCE**

1. **Before you were incarcerated, how long had you lived in your neighborhood?** _____ (months) _____ (years) (OR 777/888/999)
2. **In the neighborhood where you lived before being incarcerated, were you afraid you would be hurt by violence?**
3. _____ All the time
4. _____ Most of the time
5. _____ A little bit of the time
6. _____ None of the time

7. _____Don’t know

8. _____Prefer not to Answer

9. _____Not Applicable

1. **In the neighborhood where you lived before being incarcerated, did anyone ever use violence, such as a fight (hitting, pushing, and shoving), against you or any member of your family?**
2. _____ Yes
3. _____ No

7. _____Don’t know

8. _____Prefer not to Answer

9. _____Not Applicable

**In the neighborhood where you lived before being incarcerated, did any of the following happen in the past 6 months?**

|  | Yes  1 | No  2 | Don’t Know  7 | Prefer not to Answer  8 | NA  9 |
| --- | --- | --- | --- | --- | --- |
| 1. **A fight in which a weapon was used?** |  |  |  |  |  |
| 1. **A violent argument between neighbors or friends?** |  |  |  |  |  |
| 1. **A gang fight?** |  |  |  |  |  |
| 1. **A robbery or mugging?** |  |  |  |  |  |
| 1. **A murder?** |  |  |  |  |  |

**SOCIAL CAPITAL**

1. **How many volunteer organizations are you a member of? ____________**

(#)(OR 777/888)

1. **Generally, do you believe that most people can be trusted or that you can’t be too careful in dealing with people?**
2. _____ Most people can be trusted
3. _____ You can’t be too careful in dealing with people

7. _____Don’t know

8. _____Prefer not to Answer

9. _____Not Applicable

**How much confidence do you have in the following institutions?**

|  | A great deal  1 | Quite a lot  2 | Not vry much  3 | None at all  4 | Hard to answer 5 | Don’t Know  777 | PNA  888 | NA  999 |
| --- | --- | --- | --- | --- | --- | --- | --- | --- |
| **97.The legal system** |  |  |  |  |  |  |  |  |
| **98.The police** |  |  |  |  |  |  |  |  |
| **99.The government** |  |  |  |  |  |  |  |  |

**For the following 13 questions, please think about the neighborhood you lived in prior to your incarceration**

**100. Do you think in your neighborhood people generally trust each other in matters of lending and borrowing**?

1. _____Do trust
2. _____Do not trust

7. _____Don’t know

8. _____Prefer not to Answer

9. _____Not Applicable

**101. Suppose your neighbor suffered an economic loss, say “job loss”. In that situation, who do you think would assist him/her financially?** [Record first three mentioned.]

1. _____No one would help
2. _____Family
3. _____Neighbors
4. _____Friends
5. _____Religious leader or group
6. _____Community leader
7. _____Business leader
8. _____Police
9. _____Family court judge
10. _____Patron/employer/benefactor
11. _____Political leader
12. _____Mutual support group to which s/he belong
13. _____Assistance group to which s/he belongs
14. _____Other __________________________

77. _____Don’t know

88. _____Prefer Not to Answer

99. _____Not Applicable

**102. Do you agree or disagree that people here look out mainly for the welfare of their own families and they are not much concerned with neighborhood welfare?**

1. _____Strongly agree
2. _____Agree
3. _____Disagree
4. _____Strongly disagree

7. _____Don’t know

8. _____Prefer not to Answer

9. _____Not Applicable

|  | Strong-ly agree  1 | Agree  2 | Dis-agree  3 | Strong-ly dis-agree  4 | Don’t know  7 | Prefer not to Answer  8 | NA  9 |
| --- | --- | --- | --- | --- | --- | --- | --- |
| **103. Most people in my neighborhood are basically honest and can be trusted** |  |  |  |  |  |  |  |
| **104. People are always interested only in their own welfare** |  |  |  |  |  |  |  |
| **105. People in my neighborhood are always more trustworthy than others** |  |  |  |  |  |  |  |
| **106. In my neighborhood, one has to be alert or someone is likely to take advantage of you** |  |  |  |  |  |  |  |
| **107. If I have a problem, there is always someone to help me in my neighborhood** |  |  |  |  |  |  |  |
| **108. I do not pay attention to the opinions of others in my neighborhood** |  |  |  |  |  |  |  |
| **109. Most people in my neighborhood are willing to help if you need it** |  |  |  |  |  |  |  |
| **110. My neighborhood has prospered in the last five years** |  |  |  |  |  |  |  |
| **111. I feel accepted as a member of my neighborhood** |  |  |  |  |  |  |  |
| **112. If you drop your wallet or purse in my neighborhood, someone will see it and return it to you.** |  |  |  |  |  |  |  |

**DISCRIMINATION**

**113. How often are you discriminated against because of your race?**

1. _____Very often
2. _____Often
3. _____Every now and then
4. _____Rarely
5. _____Never

7. _____Don’t know

8. _____Prefer not to Answer

9. _____Not Applicable

**INCARCERATION HISTORY**

|  | # | Don’t Know  777 | Prefer not to Answer  888 | NA  999 |
| --- | --- | --- | --- | --- |
| **114. In your lifetime, how many times have you been arrested?** |  |  |  |  |
| **115. In your lifetime, how many months have you spent in jail or prison?** |  |  |  |  |
| **116. In the past year, how many days were you incarcerated? [0-365]** |  |  |  |  |

**PRIORITIES/CHALLENGES**

**117. Think for a minute about when you are released. What would you say are the three most difficult problems you'll face when released from jail (top three)?**

**[Show Response Card #3**]

1. _____Legal problems
2. _____Substance abuse
3. _____Housing
4. _____HIV/AIDS treatment
5. _____Pregnancy prevention
6. _____Other medical or health problems
7. _____Family problems with adults
8. _____Family problems with kids
9. _____Unemployment
10. _____Financial issues
11. _____Mental health issues
12. _____Educational
13. _____Child care
14. _____Sexual / physical abuse treatment
15. _____Sexual / physical abuse prevention
16. _____Other _____________________

77. _____Don’t know

88. _____Prefer not to Answer

99. _____Not Applicable

**THESE NEXT 11 QUESTIONS ARE TO BE ASKED OF WOMEN, ONLY.**

**118. How many Pap tests have you ever had in your life**? **____________** (#)(OR 777/888)

**119. How often do you have Pap test usually?**

1. _____Every year

2. _____Every 2-3 years

3. _____Every 5 years

4. _____Every 10 years

5. _____Never

7. _____Don’t know

8. _____Prefer not to answer

**120. Where do you go to get a Pap test usually?**

1. _____My family doctor
2. _____Health center or community clinic (not a city/county health department)
3. _____City or county health department
4. _____A hospital emergency room or clinic
5. _____Van (mobile health center)
6. _____Healer
7. _____Pharmacist
8. _____Other health care provider_____________________
9. _____ Never had a Pap test

77. _____Don’t know

88. _____Prefer not to Answer

**121. Has a doctor, nurse, or other health professional ever told you that you had an abnormal Pap result?**

1. _____Yes
2. _____No

7. _____Don’t know

8. _____Prefer not to Answer

9. _____Not Applicable

**122. If so, how many times has someone told you that you had an abnormal Pap result in your life?** **____________** (# OR 777/888/999)

**122a. The last time a doctor, nurse, or other health professional told you that you had an abnormal Pap result, what did you do?**

1. _____I got a colposcopy/biopsy
2. _____I went back to get another Pap test
3. _____I didn’t do anything

77. _____Don’t know

88. _____Prefer not to Answer

99. _____Not Applicable

**123. Has a doctor, nurse, or other health professional ever told you that you had HPV or human papilloma virus?**

1. _____Yes
2. _____No

7. _____Don’t know

8. _____Prefer not to Answer

**124. Have you ever had the HPV vaccine, Gardasil?**

1. _____Yes
2. _____No

7. _____Don’t know

8. _____Prefer not to Answer

**125. Has a doctor, nurse, or other health professional ever told you that you had cervical cancer?**

1. _____Yes
2. _____No

7. _____Don’t know

8. _____Prefer not to Answer

**126. Have you ever been treated for cervical cancer, for example, with surgery, radiation, or chemotherapy?**

1. _____Yes
2. _____No

7. _____Don’t know

8. _____Prefer not to Answer

**135. If you have taken oral contraceptives or “the pill,” for how many years did you take them?** **____________** (#, 0 Never taken OR 777/888)**Response Card #1 [for Questions 22-30]**

**Hospitals**

- - - 1. Truman Medical Center (Hospital Hill)
      2. Truman East (Lakewood)
      3. St. Luke’s Medical Center
      4. Research Medical Center
      5. KU Medical Center
      6. North Kansas City Hospital
      7. Shawnee Mission Medical Center
      8. Center Point Hospital, Independence
      9. Providence Medical Center
      10. Overland Park Regional
      11. Olathe Medical Center
      12. Menorah Medical Center
      13. St. Luke’s South
      14. Shawnee Mission South
      15. St. Joseph’s Medical Center
      16. VA Hospital

**Community Clinics**

- - - 1. KC Free
      2. SW Blvd Family Health Center
      3. Cabot Westside
      4. Swope Health Services
      5. Samuel J. Rogers
      6. Sojoiner Clinic
      7. Jackson County Free Clinic (Independence)
      8. Duchesne Clinic
      9. Swope Wyandotte
      10. Mercy & Truth (KCK)
      11. Jay Doc
      12. Health Partnership Clinic (Johnson County)
      13. Mercy & Truth (Shawnee)
      14. VA Clinic

**Health Departments**

- - - 1. KC Health Department
      2. Jackson County Health Department
      3. Wyandotte County Health Department
      4. Johnson County Health Department

**Other Mental Health and Substance Abuse Clinics**

- - - 1. CRC Health Group Kansas City Treatment Center
      2. KC Metro Methadone Program
      3. Kansas City Treatment Center
      4. Kansas Multicultural Alcohol and Drug Treatment Center
      5. Johnson County Mental Health Center
      6. Rainbow Mental Health
      7. Riverview Health Services
      8. Salvation army (KS)
      9. Salvation Army (MO)
      10. Wyandotte Center for Community Behavioral Healthcare
      11. Department of Mental Health (MO)
      12. Heart of America
      13. Kansas City Community Center
      14. Tri-County Mental Health

**“Other” or Non-response**

- - - 1. Other ______________________________

77 Don’t Know, 88 Prefer Not to Answer, 99 Not Applicable

**Response Card #2 [For Question 55**]

1. _____Condoms
2. _____Birth control pills
3. _____Other hormonal birth control methods (shot, the patch, the ring, IUD)
4. _____Withdrawal or “pulling out”
5. _____Tubal ligation or vasectomy
6. _____Not having sex during certain times of the month
7. _____Hope/prayer/luck
8. _____I didn’t use any methods to prevent pregnancy

77. _____Don’t Know

88. _____Prefer not to Answer

99. _____Not Applicable

**Response Card #3 [For Question 117**]

1. _____Legal problems
2. _____Substance abuse
3. _____Housing
4. _____HIV/AIDS treatment
5. _____Pregnancy prevention
6. _____Other medical or health problems
7. _____Family problems with adults
8. _____Family problems with kids
9. _____Unemployment
10. _____Financial issues
11. _____Mental health issues
12. _____Educational
13. _____Child care
14. _____Sexual / physical abuse treatment
15. _____Sexual / physical abuse prevention
16. _____Other _____________________

77. _____Don’t Know

88. _____Prefer not to Answer

99. _____Not Applicable
